# NOD1, NOD2, and NLRC5 Receptors in Antiviral and Antimycobacterial Immunity

**DOI:** 10.3390/vaccines10091487

**Published:** 2022-09-07

**Authors:** Magdalena Godkowicz, Magdalena Druszczyńska

**Affiliations:** 1 Lodz Institutes of the Polish Academy of Sciences, The Bio-Med-Chem Doctoral School, University of Lodz, 90-237 Lodz, Poland; 2 Department of Immunology and Infectious Biology, Faculty of Biology and Environmental Protection, University of Lodz, Banacha12/16, 90-237 Lodz, Poland

**Keywords:** NLR, antimicrobial immunity, ssRNA virus

## Abstract

The innate immune system recognizes pathogen-associated molecular motifs through pattern recognition receptors (PRRs) that induce inflammasome assembly in macrophages and trigger signal transduction pathways, thereby leading to the transcription of inflammatory cytokine genes. Nucleotide-binding oligomerization domain (NOD)-like receptors (NLRs) represent a family of cytosolic PRRs involved in the detection of intracellular pathogens such as mycobacteria or viruses. In this review, we discuss the role of NOD1, NOD2, and NLRC5 receptors in regulating antiviral and antimycobacterial immune responses by providing insight into molecular mechanisms as well as their potential health and disease implications.

## 1. Introduction

Pattern recognition receptors (PRRs) play an important role in non-specific immunity. In a sense, these receptors provide a universal alarm signal that enables the rapid transmission of danger information and thus an immediate host defense. PRRs recognize not only pathogen components, such as lipopolysaccharide, glycolipids, and nucleic acids, but also toxic metabolic products. An important class of intracellular PRRs is the NOD-like receptor (NLR) family [1,2].

## 2. The NLR Family

In humans, the NLR family includes at least 23 receptors that share a similar structure. Most proteins belonging to the NLR family contain three domains: an N-terminal signaling domain (pyrin domain (PYD) or caspase recruitment containing domain (CARD)), central nucleotide binding and oligomerization domain (NOD1 or NOD2), and C-terminal agonist sensing/binding domain (Leucine Rich Repeat (LRR)) [3]. Based on the N-terminal region, this family has been divided into four groups: (1) NLRP (NOD-like receptor P; P for PYD), (2) NLRC (NOD-like receptor C; C for CARD), (3) NLRA (NOD-like receptor A; A for the acidic transactivating domain), (4) NLRB (NOD-like receptor B; B for the baculovirus inhibitor of apoptosis (BIR)) (Table 1).

NLR receptors play a key role in regulating the host innate immune response. They recognize a variety of ligands from microbial pathogens (peptidoglycan, viral RNA, flagellin), host cells (cholesterol crystals, uric acid), and environmental sources (asbestos, silica, alum).The majority of NLRs function as PRRs, detecting specific ligands and inducing inflammatory reactions, however some NLRs do not function as PRRs but respond to cytokines, such as interferons. Inflammasome platforms formed by NLRs, which function as scaffold proteins, stimulate mitogen-activated protein kinase (MAPK) and nuclear factor-κβ (NF-κβ) signaling pathways and control the activation of inflammatory caspases. In this review, we discuss the role of three NLR molecules, NOD1, NOD2, and NLRC, in antiviral and antimicrobial immune responses, providing insight into the molecular mechanisms and their potential implications for health and disease.

### 2.1. NLRP

The most abundant group of NLRs is the NLRP subfamily, consisting of 14 proteins that are mostly composed of a PYD effector domain, a central NOD domain, and a C-terminal LRR domain. NLRP1 and NLRP10 have a different structure. NLRP1 protein has an additional finder function domain (FIND) and a caspase activation and recruitment domain (CARD), whereas NLRP10 lacks the LRR domain [4]. Recent studies have shown that autolytic proteolysis within FIND is non-specific for inflammasome activity [5], but the specific function of this domain is unknown. In contrast, CARD has the ability to attach caspases involved in inflammation and apoptosis (caspase 1) [6,7]. Many receptors in this subfamily (NLRP1, NLRP3, NLRP6, NLRP7, NRLP10, and NRLP12) are involved in inflammasome formation [8].

### 2.2. NLRC

The NLRC subfamily consists of six proteins, NOD1, NOD2, NLRC3, NLRC4, NLRC 5, and NLRX1—also named NOD5 or NOD9—and their common feature is the presence of the LRR and NOD domains. NLRX1 is unique because of the fact that, unlike the other NLRs, it is located in the mitochondria. The protein is referred to as NLRX1 since its N-terminus has not been completely defined [9]. There are different receptor structures in the NLRC subfamily. NOD1 and NLRC4 have one CARD domain and NOD2 has two CARD domains, while NLRC3, NLRC5, and NLRX1 still do not have an identified NH2 domain [10]. The best known proteins are NOD1 and NOD2, which recognize different bacterial and viral components [11]. NOD1 recognizes gamma-glutamyl diaminopimelic acid (iE-DAP), a peptidoglycan product of Gram-negative bacteria. In contrast, NOD2 recognizes muramyl dipeptide (MDP) present in the peptidoglycans of most bacteria [12,13]. Recent studies have shown that NLRs can also recognize some viruses through NOD2, which reacts with viral single-stranded RNA (ssRNA), leading to type I interferon production [14,15].

### 2.3. NLRA and NLRB

In the other two subfamilies (NLRA, NLRB), one member of each has now been identified, a class II transactivator (CITTA) and a neuronal apoptosis inhibitory protein (NAIP), respectively. What distinguishes these receptors from the others is the presence of an acidic activation domain (AD) in NLRA and BIR in NLRB [16,17]. CIITA activation is regulated by a number of post-translational modifications, such as acetylation, phosphorylation, and ubiquitination. CIITA activates MHC class II gene expression in different populations of antigen-presenting cells (APCs). In the absence of CIITA expression, MHC class II activation does not occur. CIITA is responsible for the expression of accessory proteins required for proper peptide presentation through MHC class II [16,18,19]. NAIP member, NLRB, is involved in host defence and cell survival. The characteristic feature of this protein is the inhibition of apoptosis by suppressing the activities of caspase (CASP) 3 (CASP3), CASP7, and CASP9; the autocleavage of pro-CASP9; and the cleavage of pro-CASP3 by CASP9 [16,20]. 

## 3. Structure and Expression of NOD1 and NOD2

Through sequence-homology searches, we were able to identify NOD1 and NOD2 as the first members of the NLR family. The NOD1 receptor consists of 953 amino acids and is located on chromosome 7p14-p15, while NOD2 contains 1040 amino acids and is located on human chromosome 16p21 [21,22,23]. While NOD1 shows a ubiquitous expression pattern, NOD2 is more strongly expressed in macrophages, dendritic cells, paneth cells, keratinocytes, epithelial intestinal cells, lung epithelial cells, oral epithelial cells, and osteoblasts ([24]). Both receptors recognize peptidoglycan residues, with NOD1 reacting with the peptidoglycan (PG) group containing gamma-glutamyl diaminopimelic acid (iE-DAP), which is mainly found in Gram-negative bacteria [13,25,26]. The NOD2 receptor senses MDP structures found in both Gram-positive and Gram-negative bacteria [12,27]. Recent studies suggest that NOD2 directly binds MDP with high affinity [28], and N-glycolyl MDP more strongly modulates the host response compared to N-acetyl MDP [29]. An increasing number of studies suggest that, in addition to peptidoglycan recognition, NOD1 and NOD2 may respond to danger signals or damage-associated molecular patterns (DAMPs). Endoplasmic reticulum (ER) stress contributes to inflammatory diseases such as Crohn’s disease and type 2 diabetes. ER stress induced by various microbial infections can be interpreted as a danger signal recognized by NOD1 and NOD2. *Brucella abortus* infection was shown to induce ER stress, which promoted NOD1/2-dependent inflammation and IL-6 production [30]. Furthermore, bacterial effector proteins can activate NOD1 and NOD2. *Shigella flexneri* effector proteins, such as OspB and IpgB2, and *Salmonella enterica* serotype, Typhimurium SipA and SopE, induce NF-κB activation in a peptidoglycan-independent, but NOD1- or NOD2-dependent, manner [31,32,33]. Recent studies have shown that NLRs can also recognize certain viruses through NOD2, which reacts with viral ssRNA, leading to type I interferon production [14,15]. Finally, NOD1 and NOD2 signaling leads to the activation of host defense mechanisms, such as the production of pro-inflammatory cytokines and antimicrobial molecules [34,35]. NOD1 and NOD2 are cytoplasmic receptors, but their activation depends on their location in endosomes and in the plasma membrane [36,37,38]. The recruitment of NOD1 and NOD2 to the cell membrane occurs through interaction with the actin cytoskeleton [39,40]. Rho GTPases, which are responsible for regulating the actin cytoskeleton, are known to be able to interact with and activate NOD1 [32]. Furthermore, NOD2 binds to regulators of the actin cytoskeleton, GTPase Arf [41]. Moreover, transport to the cell membrane requires lipid modification (S-palmitoylation) for recruitment to the cell membrane [42].

**Figure 1:**
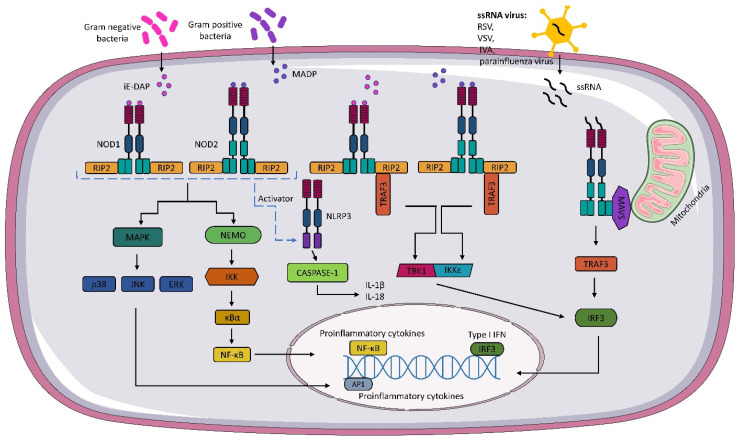
**NOD1 and NOD2 signaling pathway.** Gamma-glutamyl diaminopimelic acid (iE-DAP) and muramyl dipeptide (MDP) activate the nucleotide-binding oligomerization domains 1/2 (NOD1) and (NOD2), respectively. Activation of NODs leads to the recruitment of receptor-interacting serine/threonine kinase (RIP2). In the next step, activated RIP2 can lead to ubiquitination of the essential modulator of NF-κB (NEMO) and activation of the IKK (IκB kinase) complex. The activated IKK complex phosphorylates the inhibitor of kappaB (IκBα). The activated IKK complex phosphorylates the kappaB inhibitor (IκBα), leading to the release of NF-κB, which, after translocation to the cell nucleus, binds to kappaB (κB) elements, thereby activating pro-inflammatory cytokines. NOD1 and NOD2 also activate mitogen-activated protein kinases (MAPKs), such as p38, c-Jun N-terminal kinase (JNK), and extracellular signal-regulated kinase (ERK). NOD1 and NOD2 also interact with the NLRP3 inflammasome, which leads to caspase-1 activation and IL-18 and IL-1β production. Activation of NOD1 and NOD2 results in the formation of a TBK1 and the inhibitor of the nuclear factor kappaB kinase (IKKε) complex, which leads to the expression of type I IFNs. As well, through the interaction of NOD2 with mitochondrial antiviral signaling protein MAVS, the expression of IFN I genes occurs.

Upon recognition of the appropriate agonist by NOD1 and NOD2 receptors, activation of pathways whose primary targets are MAPK, caspase-1, and NF-κΒ occurs. These activations lead to the transcription of relevant genes and the production of inflammatory mediators. The activation of NOD1 and NOD2 signaling pathways is regulated by post-translational modifications, such as phosphorylation and ubiquitination. The recognition of iE-DAP and MADP by NOD1 and NOD2, respectively, leads to a change in receptor conformation and exposure of the CARD domain, which recruits and activates receptor-interacting serine/threonine kinase 2 (RIP2) [21,43,44]. The activation of RIP2 kinase is necessary to activate the inflammatory signaling pathway associated with NOD1 and NOD2 receptors [45]. In the next step, RIP2 can lead to the ubiquitination of the essential modulator of NF-κB (NEMO) and activation of the IKK (IκB kinase) complex. The IKK complex phosphorylates the inhibitor of kappaB (IκBα), which is then degraded, and the released NF-κB is translocated to the cell nucleus. In the cell nucleus, NF-κB dimers bind to kappaB (κB) elements, activating pro-inflammatory cytokines and factors responsible for immune cells, among others [46,47,48,49] (Figure 1).

The formation of nodosome complexes can also lead to the activation of one of the three major MAP kinase families: p38, c-Jun N-terminal kinase (JNK), and extracellular signal-regulated kinase (ERK). As a result, the MAPK pathway is activated, and p38, JNK, and ERK translocate to the nucleus and phosphorylate AP-1 transcription factors responsible for the expression of pro-inflammatory cytokines and chemokines [34,50,51] (Figure 1). 

In addition to the pathways mentioned above, NOD 1 and NOD2 receptors can also activate the interferon signaling pathway. Sabbah et al. showed that NOD2 not only recognizes MDP, but also interacts with viral ssRNA [14]. The researchers revealed that RSV activates NOD2 through the mitochondrial antiviral signaling protein (MAVS), which leads to the activation of the regulatory transcription factor, interferon 3 (IRF3). In subsequent steps, IRF3 migrates to the nucleus and activates type I IFN (IFNα/β) genes. Interestingly, the IFN pathway is also activated during bacterial infections. The mechanism is not fully understood, but it has been shown that upon binding of bacterial ligands, conformational changes of NOD1 and NOD2 occur, followed by the formation of the RIP2-TRAF3 complex, which recruits TANK shunt kinase 1 (TBK1) and the inhibitor of nuclear factor kappaB kinase (IKKε). These two kinases form a TBK1-IKKε complex that drives IRF3-dependent expression of IFNβ and type I IFN genes [52,53] (Figure 1). 

It is still not entirely clear how the NOD1 and NOD2 signaling pathways are regulated. Several studies have shown that certain molecules can positively or negatively affect NOD1 and NOD2 activation pathways. GRIM-19 protein and vimentin have been described as positive regulators of this process. They are required for NF-κB activation after MDP recognition by NOD2 [54,55]. A negative regulator of NOD signaling is the Erbin protein, which, when bound to NOD2, inhibits MDP-induced signaling [56]. Moreover, overexpression of Centaurin β-1 (CENTβ1), a GTPase-activating protein, inhibits NOD1- and NOD2-dependent expression of NF-κB [57]. 

## 4. Structure and Expression NLRC5

Among NLR members, NLRC5 contains the largest number of LRRs, consisting of 713 amino acids, making it the largest protein in this receptor family [58]. The complete human NLRC5 gene consists of 1866 amino acids and is located at locus 16q13 [59]. NLRC5 responds to viral components and lipopolysaccharides, and is highly sensitive to interferon γ (IFN-γ) [58,60,61]. NLRC5 has a structure similar to the other receptors belonging to the NLR family; however, the N-terminal CARD of NLRC5 is characterized by the folding of the death domain, making it an atypical CARD domain [62]. Furthermore, the NOD domain of the NLRC5 receptor contains the Walker A and Walker B regions, which are required for the binding and hydrolysis of nucleotide triphosphate, respectively. The Walker A motif is essential for NLRC5 migration into the nucleus and, consequently, for the transactivation activity of major histocompatibility complex (MHC) class I genes [63,64]. The structure of NLRC5 is highly conserved in many mammalian species; the homology of human NLRC5 to mice NLRC5 is 64% [65], suggesting that NLRC5 shows similar functions in different organisms.NLRC5 is highly expressed in immune cells and tissues, such as the spleen, lymph nodes, bone marrow, thymus, intestine, lung, and bone marrow [59,60,65].

**Figure 2:**
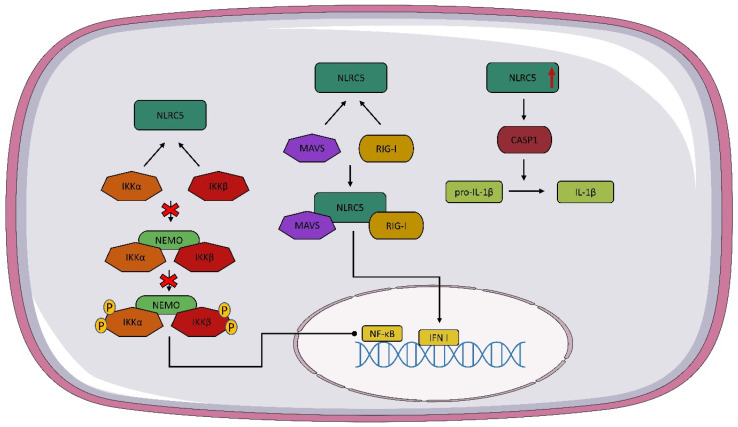
**NLRC5 signaling pathway**. As a result of the interaction of NOD-like receptor C5 NLRC5 with IκB kinase alpha/beta (IKKα/β), the NEMO IKKα/β complex is not formed and nuclear factor kappa B (NF-κB) expression is stopped. The interaction of NLRC5 with mitochondrial antiviral signaling protein (MAVS) and RIG-I leads to the formation of a complex of NLRC5 plus MAVS and RIG-I, which contributes to the expression of IFN I. Overexpression of NLCR5 activates caspase 1 (CASP1), which converts pro-IL-1β to active IL-1β.

NLRC5 has different functions depending on its localization. In the cytoplasm, NLRC5 can inhibit nuclear factor kappa B (NF-κB) signaling by interacting with IκB kinase alpha/beta (IKKα/β) so that NEMO does not bind to IKK and autophosphorylation and kinase activity are inhibited [59,65]. NLRC5 in the cytoplasm is involved in the regulation of type I IFN, but data on the effect of this receptor on IFN I are conflicting. Some studies suggest that NLRC5 interacts with RIG-I and MAVS, resulting in an enhanced IFN I response. (Figure 2) However, there are also reports suggesting that NLRC5 inhibits the IFN I response [65,66,67]. The formation of the inflammasome is important in the recognition of the infectious agent and in modulating the host immune response. It has been speculated that NLRC5 may lead to the activation of the inflammasome and even to the formation of its own inflammasome [68]. NLRC5 is involved in the activation of the NLRP3 inflammasome. The overexpression of NLRC5 leads to the increased activation of caspase-1, which converts pro-IL-1β into active IL-1β [67,68] (Figure 2). Tong et al. and Kumar et al. observed similar levels of IL-1β secretion in wild-type and NLRC5-/- mice [67,69]. Recent studies suggest that NLRC5 interacts with tumors through multiple pathways, such as β-catenin, TGF-β, and Akt [70,71,72]. It is worth noting that most of the presented functions of NLRC5 are still unclear and debatable, so further studies on this receptor are needed. 

## 5. Interaction of NOD1, NOD2, and NLRC5 with Viruses

Recent studies have shown that recognition of viral ssRNA by NOD2 leads to the activation of interferon regulatory factors (IRF) 3 and IRF 7, and the induction of type I-mediated antiviral responses [14]. Due to the lack of structural similarities between bacterial MDP and viral ssRNA motifs, the molecular recognition and signaling of the NOD2 receptor are still unclear. NOD2 is thought to be activated by direct interaction with the viral genome or viral proteins [14,73]. Several studies have revealed that in the absence of NOD2, a strong antiviral response does not occur and the control of infection and viral replication is impaired [14,74]. Therefore, it is believed that NOD2 along with the other NLRs (e.g., NOD1 and NLRC5) may promote inflammation and an antiviral response [74].

NOD2 has been shown to be involved in host defense against viral infections caused by the respiratory syncytial virus (RSV), vesicular stomatitis virus (VSV), influenza A virus (IAV), parainfluenza virus 3, and Middle East respiratory syndrome (MERS-CoV) [14,15] (Table 2). RSV infection has been found to increase the expression of NOD2, which plays a key role in the induction of IFN-β production in cells [14,75]. Once the viral ssRNA is recognized, NOD2 uses the adaptor protein, MAVS (mitochondrial antiviral signaling), to activate IRF3 (interferon regulatory factor-3) and nuclear factor kappa-light-chain-enhancer of activated B cells (NF-κB), leading to IFN production. Mice lacking NOD2 do not produce IFN efficiently and show increased susceptibility to RSV-induced pathogenesis, suggesting that this receptor plays a key role in the antiviral immune response [14]. Another study by Gao et al. revealed that RSV infection leads to the induction of NLRC5, an intracellular NOD-like protein containing the CARD domain, which has recently been identified as a transactivator of major tissue compatibility complex (MHC) class I. RSV infection of pulmonary epithelial cells was found to increase retinoic acid-inducible gene I (RIG-I) expression, resulting in NLRC5 induction and subsequent upregulation of MHC class I molecules. It is interesting to note that NLRC5 induction came after IFN-β induction and RIG-I upregulation because RIG-I suppression inhibited the expression of both NLRC5 and MHC-I [76].

The activation of the NOD2 signaling pathway is required for the production of type I IFN after infection with three other ssRNA viruses: VSV, IVA, and parainfluenza virus. Recent data suggest that direct interaction between ssRNA and NOD2 activates MAVS, leading to IRF3 activation and IFN-beta secretion [14]. During IAV infection, NOD2-/- mice were found to exhibit reduced IFN-β levels and a lower activity of dendritic cells, which were more susceptible to lung cell death. In addition, CD8+ T cell production and activation, as well as IFN-γ secretion, were shown to be lower in NOD-/- mice [77]. The results of this study suggest that NOD2 plays a key role in inducing both innate and adaptive immune responses that are required to control IVA infection. We also demonstrated an altered CD8+ T cell response during IAV infection in NLRC5-/- mice. Although reduced CD8+ T cell production was not observed in NLRC5-/- mice, IAV-specific CD8+ T cells exhibited impaired effector functions, which may contribute to the impaired clearance of viral infection [78]. 

The role of NOD2 in SARS-CoV2 recognition is not yet fully understood, but it is worth noting that there are reports that another coronavirus, MERS-CoV, is able to reduce NOD2 expression in macrophages [15]. Furthermore, infection of human fetal brain cells with Zika virus induces NOD2 expression [79]. At present, there is very little information on the role of NOD2 in SARS-CoV-2 infection. A bioinformatics analysis revealed the differential expression of NOD2 in COVID-19 patients. NOD2 expression was downregulated in patients with mild COVID-19 compared to healthy individuals. However, higher levels of NOD2 expression were observed in individuals with severe forms of the disease [80]. Interestingly, SARS-CoV-2 infection can activate NOD1 (a receptor for Gram-negative bacteria), leading to the activation of the NF-κB pathway and IFN secretion [81]. 

A great number of studies have suggested that NLRC5 may enhance the antiviral signaling that is involved in the modulation of the innate antiviral immune response [66]. It has been shown that NLRC5 efficiently inhibits viral infection by preventing the activation of RIG-I and MDA5, as well as the generation of type I IFN [82]. Nevertheless, in vitro studies have shown that NLRC5 can regulate antiviral type I IFN production either negatively or positively, suggesting a contradictory antiviral function of NLRC5 [58,60,65]. NLRC5 is an important regulator of MHC class I expression that mediates antiviral activity, SARS-CoV-2, like other viruses, has evolved mechanisms for targeting the MHC class I pathway to evade host immunity. In nasopharyngeal samples from SARS-CoV-2 patients, it was observed that NLRC5 expression was downregulated or not induced at all. The Open Reading Frame 6 (ORF6) SARS-CoV-2 protein has been shown to reduce IFN-γ-mediated NLRC5 expression and inhibit nuclear transport of NLRC5 [83].

## 6. Interaction of NOD1, NOD2, and NLRC5 with Mycobacteria

As receptors that recognize peptidoglycan fragments, NOD1 and NOD2 are known to be involved in the response to many bacterial infections. NOD1 aids in the immune clearance of *Shigella flexneri*, *Escherichia coli*, *Helicobacter pylori*, *Pseudomonas aeruginosa*, *Campylobacter jejuni*, *Legionella pneumophila*, *Clostridium difficile*, and *Haemophilus influenzae*, leading to the activation of the NF-κB pathway [84,85,86,87,88,89,90]. NOD2 is involved in the recognition of bacterial pathogens such as *Streptococcus pneumoniae, Listeria monocytogenes, Salmonella enterica* Typhimurium, *Shigella flexneri,* and *Mycobacterium tuberculosis* [34,91,92,93]. There are increasing numbers of reports on the role of NOD1 and NOD2 in activating the adaptive immune response. NOD2 stimulation by MDP has been shown to trigger a strong antigen-specific Th2 immune response, resulting in IL-4 and IL-5 production and IgG1 responses [94].

NOD2 plays a key role in host innate immunity to bacterial lung infection [95,96,97,98]. Interactions between pathogenic mycobacterial cell elements and NOD2 receptors play a key role in the course of infection. NOD2 regulates the production of inflammatory mediators in response to MDP, which is present in the *Mycobacterium* cell wall [12,29]. The role of NOD2 in mycobacterial infection has been mainly studied in *Mycobacterium tuberculosis*(*M.tb*) and *Mycobacterium bovis* BCG (BCG) infection models. Previous studies have shown that impaired NOD2 signaling results in inappropriate recognition of mycobacteria in vitro by macrophages [91,99,100,101]. The stimulation of human macrophages with NOD2 ligands led to the secretion of more TNF-α and IL-1β in response to *M.tb* and BCG [102]. Furthermore, NOD-deficient mice showed reduced TNF-α and NO production in response to *M.tb* [91,99]. It is supposed that if the initial host defense mechanisms fail, then NOD2 is a secondary line of defense for macrophages and, more importantly, mediates iNOS production, which leads to NO secretion in response to *M.tb* [103]. NOD1-deficient mice were shown to respond normally to *M.tb* infection, and NOD1 alone could not compensate for the lack of NOD2 [91]. Little is known about the role of NOD2 in adaptive immunity to mycobacteria, but Divangahi et al. demonstrated that T cell-dependent immunity was reduced in NOD2-deficient mice after mycobacterial infection of both the lung and skin [104]. In addition, we observed that the stimulation of NOD2 by MDP in human alveolar macrophages infected with *M.tb* led to the increased intracellular control of *M.tb* growth and transport to the autophagosome of proteins that are associated with autophagy [98]. Interestingly, three common non-synonymous polymorphisms in the NOD2 gene were shown to be associated with genetic susceptibility to tuberculosis in African Americans [105].

The activation of NOD1 and NOD2 can also lead to the initiation of autophagy, which relies on cooperation with the autophagy-related 16-like 1 (ATG16L1) protein. Autophagy is a highly-conserved process of cellular homeostasis, during which damaged organelles, protein aggregates, and various pathogens are removed. It has been shown that *M.tb*-infected human follicular macrophages leads to NOD2 activation, which contributes to the recruitment of the autophagy markers IRGM(immunity-related GTPase family M protein), LC3 (microtubule-associated protein 1A/1B-light chain 3), and ATG16L1 (autophagy related 16 like 1) to *M.tb*-containing vesicles. This finding demonstrates that the active NOD2 receptor in human alveolar macrophages may represent an early innate control of primary *M.tb* infections [98]. NOD1 and NOD2 are also involved in the formation of reactive oxygen species (ROS). Dual oxidase 2 (DUOX2) protein during bacterial infection has been shown to be involved in NOD2-dependent ROS production [106]. 

Referring to the study by Kleinnijenhuis et al. it can be speculated that NOD2 mediates TB resistance by modulating the innate immune response to the Bacillus Calmette-Guérin (BCG) vaccine. In this study, BCG was shown to induce NOD2-dependent epigenetic reprogramming of monocytes [107]. Arts et al. confirmed the induction of trained immunity through NOD2 receptor activation in response to γ-irradiated BCG (γBCG). NOD2-deficient patients exhibited impaired induction of trained immunity [108]. Wannigama and Jacquet suggested that BCG induces NOD2- dependent trained immunity, which may influence the course of SARS-CoV-2 infection [109]. IFN-I is known to be an important mediator of the antiviral response; however, a delayed IFN-I response may be associated with a severe course of COVID-19 disease [110]. The early induction of IFN-I and the other cytokines, followed by a slow-down in this antiviral response, may impact disease outcome. Through NOD2 signaling, BCG induces the secretion of IFN-I [111]. Hilligan et al. showed that prior intravenous injection of BCG to mice inhibited the SARS-CoV-2-induced cytokine storm. [112];this suggests that pro-inflammatory cytokine signaling might be a mechanism by which intravenous BCG injection provides protection against SARS-CoV-2. This study revealed a non-specific protective effect of the BCG vaccine against SARS-CoV-2.

Kang and Chae investigated the role of NOD receptors in *M. leprae* infection. They showed that *M. leprae* stimulated NF-κB activation and IL-1β and TNF-α expression in cells transfected with a NOD1 or NOD2 expression plasmid. A minimal response to *M. leprae* was observed in NOD1-transfected cells, which is in contrast to NOD2-transfected cells where the response was much higher [113]. This suggests that the host response to *M. leprae* is dependent on both NOD1 and NOD2. It is worth noting that *M. leprae* has a unique MDP structure, which differs from that of other mycobacteria [114]. The unique structure of *M. leprae* has been shown to induce NOD2-dependent IL-32 production and dendritic cell differentiation [115].

Lee et al. evaluated the role of NOD2 in innate host immunity to *Mycobacterium abscessus* infection. They demonstrated that NOD2 plays a key role during clearance of *M. abscessus* bacteria. NOD2-/- mice exhibited a higher bacterial load and reduced levels of IL-6, TNF-α, and IL-1β. Similar to *M. tuberculosis* infections, NOD2 was also involved in iNOS expression and NOD production in *M. abscessus*-infected macrophages [116]. In the subsequent study, Ahn et al. showed that NOD2 mediated the expression of type I IFN, which mediates NO production in *M. abscessus*-infected macrophages to provide intracellular control of mycobacterial growth [117].

Carvalho et al. investigated the role of NOD2 during infection with *Mycobacterium avium*. They found that NOD2 was not required for host immunity to *M. avium* infection, although reduced IL-12 and TNF-α secretion was observed in NOD2-deficient macrophages. Furthermore, during chronic infection, NOD2-/- mice were characterized by impaired IFN-γ production and reduced size and number of lymphocyte/granulocyte liver granulomas [118]. Ferwerda et al. first demonstrated that NOD2 was involved in the recognition of *M. avium* subsp. *paratuberculosis* [119].

In vivo studies have revealed that NLRC5 plays an important role in host defence against intracellular pathogens. It has been shown that the absence of NLRC5 reduces MHC class I expression, but does not affect MHC class II levels. In mice infected with *Listeria monocytogenes* with an NLRC5 defect, lack of activation of CD8+ T cells was observed, leading to increased bacterial load [120,121]. 

## 7. NOD1, NOD2, and NLRC5 Gene Polymorphisms and Disease Predisposition

Many pathological processes originate in defective PRR signaling, which is caused by mutations within the encoding genes. Dysregulation of NLR receptor function has been described in many diseases, including chronic inflammation, auto-immunity, and cancer [122,123]. NOD2 is one of the NLR genes that have been extensively studied in relation to the innate immune response. Polymorphisms in the gene encoding NOD2 have been linked to a growing number of chronic inflammatory disorders, such as Crohn’s disease, Blau syndrome, and early-onset sarcoidosis [124,125,126]. Three major NOD2 variants, R702W, G908R, and L1007fsinsC, and some minor variants in the C-terminal LRR region and the helix domain 2 (HD2) have been found to be associated with the development of Crohn’s disease in both European and American populations [124,125,127]. The R702W and G908R polymorphisms are single amino acid changes within the LRR domain, while the L1007fsinsC variant is caused by a deletion that leads to the loss of 33 amino acids. The relative risk of developing Crohn’s disease in homozygous or heterozygous individuals has been estimated to be between 10- and 40-times higher than that in the general population. [128]. The loss of NOD2 function resulting from these mutations enables bacterial invasion and an abnormal mucosal immune response is the underlying cause of Crohn’s disease. The inability of MDP to activate forms of NOD2 carrying mutations associated with Crohn’s disease has been observed in both in vitro and in vivo studies [124,125,129,130,131]. Mutations associated with Blau syndrome are located in the NOD domain of NOD2, and at least 17 different mutations have been identified, with the following missense mutations being the most abundant: R334Q, R334W, and L469F, which together account for 80% of cases, and the E383K variant accounting for 5% of cases [132]. These mutations have been found to cause excessive activation of NF-κB and MAPK compared to the wild-type NOD2, leading to the hypothesis that Blau syndrome is the result of gain-of-function NOD2 [126]. Genetic studies also indicate the importance of NOD2 polymorphic alleles in association with the risk of tuberculosis caused by mycobacterium tuberculosis infection [105,133]. In an African-American study, Austin et al. observed that the three common non-synonymous single-nucleotide NOD2 polymorphisms, Pro268Ser, Arg702Trp, and Ala725Gly, were significantly associated with susceptibility to active tuberculosis [105]. Moreover, in the Chinese Han population, another genetic polymorphism, Arg587Arg (CGT → CGG), located in exon 4 of the NOD2 gene, has been shown to be a possible risk factor for tuberculosis [134]. Leprosy caused by *M. leprae* is the most common mycobacterial disease associated with NOD2 polymorphisms [96,135,136,137,138]. During genome analysis of leprosy patients, Zhang et al. showed a single nucleotide polymorphism in the NOD2 gene that is associated with susceptibility to *M. leprae* infection [135]. In a Chinese population, Pan et al. also observed that variants in the NOD2 gene were significantly associated with susceptibility to leprosy [136]. Leturiondo et al. confirmed that NOD2 plays a major role in resistance against *M. leprae* in an Amazonian admixed ethnic population [138]. Interestingly, Crohn’s disease susceptibility genes (*NOD2,TNSF15*) have been shown to be associated with leprosy in a Vietnamese population [137].

It has been revealed that certain genetic NOD1 polymorphisms are associated with a susceptibility to bronchial asthma, atopic dermatitis, and inflammatory bowel disease. An insertion-deletion polymorphism (ND1+32,656) near the start of intron IX has been found to be associated with high IgE levels, while the deletion allele of a complex functional NOD1 indel polymorphism (ND1+32,656 * 1) is significantly correlated with early-onset inflammatory bowel disease [139,140].

A few studies have shown that polymorphisms in the NLRC5 gene can affect susceptibility to infection or be a determinant of chronic inflammation status [141,142]. Zupin et al. observed that the rs289723 polymorphism, located in NLRC5 gene, was closely associated with an increased risk of chronic mild periodontitis and chronic local periodontitis [141]. In addition, Zhong et al. found an association between NLRC5 allelic variants and susceptibility to pulmonary aspergillosis [142].

## 8. Potential Use of NOD1 and NOD2 Signaling Modulation in Therapy

As one of the main functions of NLRs is to detect pathogens and activate signaling pathways involved in immune processes and tissue repair, they play a key role in maintaining health and resistance to infection. Therefore, controlled modulation of the innate immune response triggered by these molecules may be a potential target for therapeutic interventions. Since NOD1 and NOD2 directly activate the NF-κB pathway, NOD1 and NOD2 signaling inhibition may be useful in treating a variety of acute and chronic disorders where the suppression of the pro-inflammatory response is desirable. NOD proteins can be modulated by several natural agents with anti-inflammatory properties. The polyphenol curcumin, which is found in the Curcuma longa plant, has been demonstrated to inhibit both lauric acid- and MDP-induced NOD2 signaling, which, in turn, inhibits NF-κB activation and IL-8 production [143]. Similar inhibitory effects on NF-κB activation have also been found for parthenolide, helenalin, and several other sesquiterpene lactones, but their mechanism of action has yet to be elucidated [144,145]. Docosahexaenoic acid and eicosapentaenoic acid are the polyunsaturated fatty acids that have the largest inhibitory effects on NOD1 and NOD2 by preventing NOD2 from self-oligomerizing, which suppresses NF-κB activation and IL-8 production [146]. In the search for small molecule NOD inhibitors, a thorough high-throughput screening (HTS) program was carried out under the supervision of the Molecular Libraries Probe Production Center Network [147,148]. Using cell-based NF-κB driven luciferase reporter gene activity to measure NOD1 modulation, several active scaffolds were found from a library of 290,000 compounds from the Molecular Libraries Small Molecule Repository of the NIH (National Institutes of Health). The indoline, tetrahydroisoquinoline, and benzimidazole scaffolds met the screening criterion of the half-maximal inhibitory concentration (IC50) values below 10 μM. Two molecules, ML130 (CID-1088438) and ML146 (CID-5310346), were identified as active NOD1-selective inhibitors, whereas a benzimidazole diamide compound designated GSK669 selectively inhibited an MDP-stimulated, NOD2-mediated IL-8 response without directly inhibiting RIP2 kinase activity [147,148,149]. In most cases, it is still unclear how these small molecules influence NOD1 and NOD2 signaling. It is possible that they act directly to affect NOD protein stability, ligand recognition, ATP binding, and hydrolysis activity, or target any of the numerous regulatory proteins in signaling pathways [150]. Although NOD1 and NOD2 are among the most thoroughly studied proteins in the NLR family, their therapeutic potential for modulation of NOD1 and NOD2 has remained largely untapped. Developing pharmacological treatments that target NOD signaling require further in-depth understanding of the mechanisms underlying NOD activation and modulation.

## 9. Future Directions and Conclusions

Although NOD-like receptors were first identified more than a decade ago, research on this specific family of microbial detectors continues to be conducted to better understand the mechanisms underlying ligand detection, signal transduction, and activation of inflammatory signaling pathways. Of great interest is the development of small-molecule antagonists that inhibit NLR activation by directly targeting specific receptor domains, such as the CARD domains in NOD1 and NOD2. New specific modulators of NLR signaling are urgently needed, as therapeutic intervention of clinical NLR dysfunction is currently only possible through the administration of potent anti-inflammatory drugs with a wide range of serious side effects. However, it must be emphasized that the strategy of using NLR signaling modulation to treat pro-inflammatory diseases and cancer requires intensive research aimed at determining the negative and positive effects of targeting receptor pathways to balance the inhibition of harmful inflammation with immune suppression and susceptibility to infection. The number of NLRs and their diversity testify to the enormous potential of these structures, which are, so far, completely untapped in animal and human clinics. The apparent rapid increase in the number of studies on NOD1, NOD2, and NLRC5 receptors in recent years suggests that the NLR family of proteins represents a huge cargo of potential biological novelties and applications.

## Figures and Tables

**Table 1:** Structures of the NLR subfamilies.

NLR Family	Member	Stucture	Reference
NLRA	CIITA	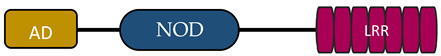	[16,17]
NLRB	NAIP	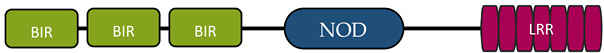	[16,17]
NLRC	NOD1	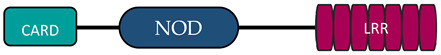	[9,10]
	NOD2	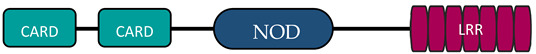	[9,10]
	NLRC4	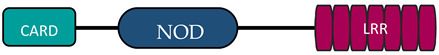	[9,10]
	NLRC3/C5/X1	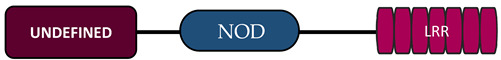	[9,10]
NLRP	NLRP1	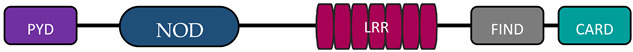	[4]
	NLRP2-9, 11-14	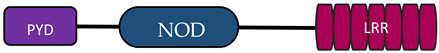	[4]
	NLRP10	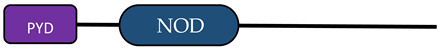	[4]

Abbreviations: AD, acidic activation domain; BIR, baculovirus inhibitor of apoptosis; CARD, caspase recruitment containing domain; CIITA, class II transactivator; FIND, finder function domain; NAIP, neuronal apoptosis inhibitory protein, NLRA, NOD-like receptor A; NLRB, NOD-like receptor B; NLRC, NOD-like receptor C; NLRP, NOD-like receptor P; NOD, nucleotide-binding oligomerization domain; LRR, leucine rich repeat; PYD, pyrin domain.

**Table 2:** Viruses stimulating NOD1, NOD2, or NLRC5 signaling.

Viruses	Receptor	Potiential Signaling Pathways	Refs
Respiratory Syncytial Virus	NOD2	MAVS-IRF3-IFN pathway	[14]
Parainfluenza Virus 3	NOD2	MAVS-IRF3-IFN pathway	[14]
Influenza A Virus	NOD2	MAVS-IRF3-IFN pathway	[14]
	NLRC5	NLRC5-RFX-CREB1-ATF1-NF-Y-X1/2-Y-MHCI pathway	[77]
Vesicular Stomatitis Virus	NOD2	MAVS-IRF3-IFN pathway	[14]
Middle East Respiratory Syndrome	NOD2	MAVS-IRF3-IFN pathway	[15]
Zika Virus	NOD2	Unknown	[79]
SARS-CoV-2	NOD1	LGP2+MDA5-MAVS-IRF3/5-IFN pathway	[81]
	NLRC5	STAT1-IRF1-NLRC5 pathway	[83]

Abbreviations: ATF1, activating transcription factor 1; CREB1, cAMP responsive element binding protein 1; IFN, interferon; IRF3, regulatory transcription factor interferon 3; NF-Y, nuclear transcription factor Y; MAVS, mitochondrial antiviral signaling protein; MDA5, melanoma differentiation-associated protein 5; MHC I, major histocompatibility complex class I; RFX, regulatory factor X.

## Data Availability

The data presented in this study are openly available under reference numbers.

## Abbreviations

AD: activation domain; APC, antigen presenting cells; BCG, Bacillus Calmette-Guerin; CARD, caspase recruitment containing domain; CASP, caspase; CENTβ1, centaurin β-1; CIITA, class II transactivator; DAMP, damage-associated molecular patterns; ERK, extracellular signal-regulated kinase; FIND, finder function domain; iE-DAP, gamma-glutamyl diaminopimelic acid; IκBα, inhibitor of kappa B; IKK, IκB kinase; IKKε, inhibitor of nuclear factor kappaB kinase; IRF3, transcription factor interferon 3; IVA, influenza A virus; JNK, Jun N-terminal kinase; LRR, leucine rich repeat; MAPK, mitogen-activated protein kinase; MDP, muramyl dipeptide; MERS-CoV, Middle East respiratory syndrome; MHC, major histocompatibility complex; *M.tb*, *Mycobacterium tuberculosis*; NAIP, neuronal apoptosis inhibitory protein; NF-κβ, nuclear factor-κβ; NLR, NOD-like receptor; NLRX1, NLR family member X1; ORF6, open reading frame 6; PG, peptidoglycan; PRR, pattern recognition receptor; PYD, pyrin domain; ssRNA, single stranded RNA; RIP2, receptor-interacting serine/threonine kinase; RSV, respiratory syncytial virus; TNSF15, tumor necrosis factor ligand superfamily member 15; VSV, vesicular stomatitis virus.
